# Association Between Knowledge of and Attitudes Toward Ageing in Kuala Lumpur: The Moderating Role of Exposure to Older Adults with Dementia

**DOI:** 10.3390/healthcare13111234

**Published:** 2025-05-23

**Authors:** Ponnusamy Subramaniam, Nor Afifah Aziz, Hend Faye AL-shahrani, Mohammad Ahmed Hammad, Muhamad Faisal Ashaari, Tay Kok Wai, Shobha Sharma

**Affiliations:** 1 Centre for Health Ageing & Wellness (HCARE), Faculty of Health Sciences, Universiti Kebangsaan Malaysia, Jalan Raja Muda Abdul Aziz, Kuala Lumpur 50300, Malaysia; ponnusaami@ukm.edu.my (P.S.); n.afifahaziz96@gmail.com (N.A.A.); 2 Department of Social Planning, College of Humanities and Social Sciences, Princess Noura bint Abdul Rahman University, P.O. Box 84428, Riyadh 11671, Saudi Arabia; hfalshahrani@pnu.edu.sa; 3 Faculty of Education, Assiut University, Assiut 71515, Egypt; mohammed.hammad@edu.aun.edu.eg; 4 Faculty of Islamic Studies, Universiti Kebangsaan Malaysia, Bangi 43600, Malaysia; faisal@ukm.edu.my; 5 Department of Psychology and Counseling, Faculty of Arts and Social Science, Tunku Abdul Rahman University, Kampar 31900, Malaysia; taykw@utar.edu.my

**Keywords:** dementia awareness, ageing, exposure, knowledge of ageing, attitudes towards ageing

## Abstract

**Background/Objectives:** Exposure to older adults has been shown to influence the formation of attitudes toward this demographic. This raises the question of whether such exposure affects the relationship between the knowledge of ageing and attitudes toward ageing. The current study aimed to assess the level of knowledge and attitude toward ageing, as well as to examine the moderating effect of exposure towards older adults. **Methods**: A cross-sectional study using convenience sampling was conducted in Kuala Lumpur and involved 392 participants with a mean age of 28.69 years (S.D. = 7.61) and an age range of 18 to 59 years. Data were collected using a questionnaire that included sociodemographic questions, Kogan’s Attitude toward Old People (KAOP), and Palmore’s Facts on Ageing Quiz (FAQ). Hierarchical regression and moderation analyses were conducted using SPSS version 23 and the PROCESS macro. **Results**: The findings show that the public had a moderate level of knowledge about ageing and a slightly positive attitude toward it. Knowing someone with dementia significantly moderated the relationship between ageing knowledge and positive attitudes toward ageing. Furthermore, the positive impact of ageing knowledge decreased as experience in caring for individuals with dementia increased. **Conclusions**: Understanding the moderating effect of caregiving for those with dementia can inform public health strategies and caregiver support programs, encouraging a more nuanced approach to ageing education that considers the practical experiences of those who interact with older adults with cognitive impairments.

## 1. Introduction

The older population is projected to grow significantly, reaching 2 billion by 2050, which represents a 50% increase globally. The majority of older adults are expected to reside in low- and middle-income countries [1]. In Malaysia, the percentage of individuals aged 65 and older rose from 6.7% to 7.0% (approximately 3.5 million people) between 2019 and 2020 [2]. Advancing age is a significant risk factor for the development of cognitive disorders, including dementia and mild cognitive impairment (MCI). Dementia is a growing public health concern in Malaysia, with projections indicating a significant increase in cases over the coming decades. In 2019, approximately 142,000 Malaysians were living with dementia, and this number is expected to rise to between 411,000 and 596,000 by 2050 [3]. This rate is notably higher in individuals with no formal education, older age, female gender, and poor self-rated health [4]. This demographic shift raises concerns about ageism, which manifests in behaviors such as avoiding interactions with older adults [5] and using patronizing language towards them [6].

The empirical literature shows inconsistent results regarding the relationship between knowledge of ageing and perceptions of older adults. Some studies focusing on this relationship reveal an inverse connection, indicating that greater knowledge of ageing correlates with lower levels of ageism [7,8]. This suggests that individuals with more knowledge about ageing tend to hold fewer negative attitudes towards older adults. For instance, Burnes et al. [9] found that people’s knowledge about ageing was linked to their attitudes towards older adults. Similarly, Stewart et al. [10] discovered that dental students with accurate knowledge about ageing exhibited more positive attitudes, while those with inaccurate knowledge had negative attitudes [11].

Conversely, some research indicates no relationship between individual knowledge of ageing and feelings towards older adults. Similar findings by Dourado et al. [12] and Stubblefield et al. [13] suggest that despite increased knowledge scores about ageing among college students, negative biases towards ageing did not improve. Other studies also indicate a lack of association between knowledge of ageing and attitudes towards older people, suggesting that attitudes towards older individuals are independent of one’s understanding of ageing [11,14].

An early study by Caspi [15] proposed that direct contact between younger and older individuals could foster more positive attitudes towards ageing among younger people. Prior research has shown that interactions with older adults are linked to ageism, which can negatively impact overall attitudes towards ageing [9,11,16]. Contact with and exposure to older adults can occur in various contexts, including personal relationships, such as having older family members, or through experiences in caregiving, education, or professional settings [17,18,19]. For example, Smith et al. [19] found that approximately 37% of participating students interacted with older adults at least once a week, 38.3% had lived with an older adult at some point in their lives, and 78.2% had volunteered or worked with an older adult. Participants who had more frequent interactions with older adults and those who had experience living with an older adult reported significantly lower negative ageist attitudes.

Moreover, interactions with older adults can vary in frequency and intimacy. Building intergenerational relationships among young, middle-aged, and older individuals is essential for fostering mutual respect, developing cooperative relationships, and revitalizing positive attitudes towards one another [20,21]. Some studies suggest that insufficient intergenerational interaction between younger individuals and older adults is a barrier to developing positive attitudes, particularly among children [22,23,24]. Conversely, Drury et al. [25] found no significant differences in attitudes towards ageing among undergraduate students based on their level of intergenerational contact, whether defined by living with older family members or interacting frequently with older adults at work. A possible explanation for the lack of a relationship between intergenerational contact and attitudes towards ageing is that the quality of interactions with older adults may be a better predictor of attitudes than the frequency of contact [26,27].

Recent initiatives aimed at improving intergenerational relations include structured programs implemented in residential care settings, such as intergenerational learning activities, shared arts programs, and volunteer programs where younger individuals engage with older adults to promote mutual understanding and reduce ageist attitudes. Furthermore, co-living programs, such as university initiatives that pair students with older adult residents in exchange for reduced housing costs, have shown promise in promoting positive interactions and mutual support. These programs demonstrate that intentional, quality contact can significantly improve younger individuals’ perceptions of ageing and provide practical models for fostering intergenerational solidarity [22,28].

Some studies have found that negative attitudes can develop from direct contact with older adults [27,29,30,31]. These findings suggest that direct contact may not always lead to positive attitudes, as the quality and nature of intergenerational interactions also play a significant role. This raises the question of whether experiences in caring for healthy or ill older adults shape different attitudes towards ageing. Despite varying findings, we hypothesize that contact with older adults with dementia will significantly and positively moderate the relationship between knowledge and attitudes toward ageing. Previous research has indicated that contact or exposure to older adults can shape attitudes towards them [25]. However, there is no clear evidence on whether this exposure moderates the relationship between knowledge and attitudes towards ageing.

Exposure to older adults with dementia may play a moderating factor because prior contact and familiarity can significantly shape individuals’ perceptions, attitudes, and behavioral responses. Research has shown that personal experience—such as knowing or caring for someone with dementia—can reduce stigma [32], enhance empathy and the willingness to engage [33], and positively influence both knowledge and behavioral intentions [33,34]. Including this factor allows for a more nuanced understanding of how contact experience may influence participants’ reactions and responses in the context of dementia-related issues.

### Theoretical Framework

#### The Contact Hypothesis

The contact hypothesis, first proposed by Allport [34], suggests that intergroup contact can reduce negative attitudes towards different groups. This theoretical framework underpins the current research, which aims to explore whether contact with older adults influences attitudes towards them. A previous study by Teater and Chonody [35] found that contact between children and older adults, as well as the age at which one considers a person to be “old”, significantly predicted positive attitudes toward older individuals. Additionally, research by Steward [36] indicated that young people in workplaces with regular interactions with older adults exhibited more positive attitudes than their peers without such exposure. However, Drury et al. [25] noted that individuals living with older adults often developed more negative attitudes compared to those who did not share a living space with them. This suggests that the formation of attitudes towards older adults through intergenerational contact may depend on the quality and nature of the interactions. For instance, young people in work settings may view older adults who seek services as competent individuals, while those living with older adults are more likely to interact with individuals who require assistance, such as those needing help with medication, suffering from dementia, or needing close monitoring [25].

Therefore, the current study aimed to (1) assess the level of knowledge and attitudes toward ageing among adults in Kuala Lumpur; (2) examine the influence of sociodemographic variables on knowledge and attitudes toward ageing; and (3) investigate whether exposure to older adults, particularly those with dementia, moderates the relationship between knowledge of ageing and attitudes toward ageing. It is anticipated that exposure to older adults, such as having experience caring for them, caring for those with dementia, or knowing someone with dementia, will significantly moderate the relationship between knowledge of ageing and attitudes toward ageing. Exposure to older adults will be assessed using participants’ demographic data, which include (1) current or past experience caring for older adults, (2) knowing someone with dementia, and (3) current or past experience caring for older adults with dementia. To address these objectives, a cross-sectional survey design was employed, and details of the methodology are outlined below.

## 2. Materials and Methods

### 2.1. Study Design

This study employed a quantitative cross-sectional design, using a convenience sampling technique to recruit participants from various public settings in Kuala Lumpur.

### 2.2. Participants and Study Location

A total of 400 participants aged 18 to 59 years old were recruited from Kuala Lumpur. Data were collected through physical surveys using structured questionnaires. Participants were recruited from public places, such as shopping malls and social events, to ensure a diverse and voluntary respondent pool. Kuala Lumpur was selected as the study location because is a highly urbanized and diverse city, making it an ideal setting for recruiting a broad and varied participant pool. Additionally, the city’s public places served as convenient venues for data collection through physical surveys, allowing for an efficient recruitment of participants.

The inclusion criteria consisted of individuals aged 18 to 59 who are Malaysian and reside in Kuala Lumpur. The exclusion criteria included (1) an inability to provide informed consent, (2) an incomplete completion of the survey instrument, and (3) non-Malaysians.

The age range was chosen based on both theoretical considerations and practical sampling strategies. Theoretically, individuals aged 18 to 59 represent the primary working-age and caregiving population and are likely to have direct or indirect exposure to older adults and dementia-related experiences, thus making their knowledge and attitudes toward ageing particularly relevant for this study. Furthermore, this age group spans young adulthood to late middle age, allowing us to explore a wide range of perceptions that may evolve with age and life experience. Practically, including participants up to 59 years helped ensure adequate sample diversity while excluding older adults themselves to avoid overlap between participant and target populations (i.e., attitudes toward older adults). Convenience sampling in public areas also made this age range accessible and feasible for recruitment.

### 2.3. Measures and Study Variables

#### 2.3.1. Demographic Data

The demographic data assessed included date of birth, age, gender, career background—specifically the scope of the career—and information regarding whether the respondent was a student, including details about their study program. Additionally, we evaluated the respondents’ experience with older adults and their prior knowledge of dementia through specific questions outlined below. Respondents indicated “Yes”, “No”, or “Not Sure” for each question. Those who answer “No” or “Not Sure” were categorized as having no experience with older adults and no prior knowledge of dementia. In contrast, respondents who answered “Yes” were categorized as having experience with older adults and prior knowledge of dementia. The questions were as follows:

1. Do you have previous or current experience in taking care of older adults?
2. Do you know someone with dementia or memory difficulties?
3. Do you have past or current experience caring for older adults with dementia?
4. Do you have formal or informal knowledge of dementia? This variable was collected for descriptive purposes only and was not included in the final analyses.

#### 2.3.2. Facts on Ageing Quiz (FAQ)

The Facts on Ageing Quiz (FAQ) [37] consists of 25 True/False statements that address basic physical, mental, and social aspects of ageing, as well as common misconceptions about it. Participants earned one point for each correct answer and received zero points for incorrect or unanswered questions. Scores ranged from 0 to 25, with higher totals indicating greater knowledge of ageing. Sample questions include, “All five senses do tend to decline in old age” and “Clinical depression occurs more frequently in older than younger people”. According to Bleijenberg [38], the scores are classified as follows: 0–8 (Low), 9–17 (Moderate), and 18–25 (High). The FAQ is recognized as a valuable tool and has been utilized in over 100 studies [39,40,41,42]. The initial study by Palmore [37] reported a Cronbach alpha of 0.28 for the FAQ1. While the psychometric properties of the FAQ have faced criticism, there is a consensus that it is a reliable measure of knowledge about ageing, with its validity and reliability empirically documented [42]. For this study, we used the Malay version of the Facts on Ageing Quiz (FAQ), which has been previously translated and validated, demonstrating good face validity, by Singh et al. [43].

#### 2.3.3. Kogan’s Attitude Towards Older People (KAOP)

Kogan’s Attitude towards Older People (KAOP) [44] consists of 34 items, including 17 positive and 17 negative statements about older adults. Respondents answered each item using a six-point Likert scale, ranging from one (strongly disagree) to six (strongly agree). To calculate total scores, the responses to negatively worded items were reversed. The total score can range from 34 to 204, with higher scores indicating more positive attitudes towards older adults. Additionally, the KAOP yields two subscales: the Positive Subscale (KAOP + ve), which includes 17 positive statement items, and the Negative Subscale (KAOP –ve), derived from 17 negative statement items about older adults. Sample questions include, “Most older people get set in their ways and are unable to change” and “Most older people are capable of new adjustments when the situation demands it”. In this study, the internal consistency, measured by Cronbach’s alpha, was α = 0.8. The questionnaire was translated into Malay, with both forward and backward translations conducted by an independent translator to ensure the validity of the translated version.

### 2.4. Procedure

Data were collected over a four-month period from December 2021 to March 2022. All respondents were recruited from various settings, including shopping malls, community centers, and other both public and private sectors in Kuala Lumpur. After screening based on inclusion and exclusion criteria, the selected respondents received an information sheet and verbal explanation of this study before signing a consent form. Following this, respondents completed a questionnaire that included sociodemographic questions, Palmore’s Fact Ageing Quiz (FAQ) [37], and Kogan’s Attitudes toward Older People (KAOP) [44]. Trained research assistants administered the questionnaires in a paper-based format during in-person sessions. Research assistants were available to address any questions during the process.

### 2.5. Data Analysis

Data were analyzed using the Statistical Package for Social Science (SPSS Version 23). Missing values for the FAQ and KAOP measurements were replaced with the mean values for each respective measure. Demographic data and total scores for FAQ and KAOP were reported descriptively. Pearson and Point Biserial correlational analyses were conducted to examine the relationships between demographic variables and the continuous variables used in this study. Exposure was operationalized as a binary variable. Participants who answered ‘Yes’ to questions regarding current or past caregiving for older adults, knowledge of someone with dementia, or caregiving experience for older adults with dementia were coded as ‘1’ (Exposure). Those who answered ‘No’ or ‘Not Sure’ were coded as ‘0’ (No Exposure).

To explore the moderating effect of exposure to older adults on the relationship between knowledge of ageing and attitudes towards ageing, a moderated multiple regression analysis was performed using hierarchical multiple regression and Process [45]. A power analysis was conducted using G*Power 3.1. With a sample size of 384 participants, a power of 0.95 was achieved for detecting a small-to-medium effect size (f^2^ = 0.05) at a significance level of α = 0.05.

Before conducting hierarchical regression and moderation analysis, assumptions of multiple regression were tested. Linearity and homoscedasticity were assessed by examining scatterplots of standardized residuals, which showed no major violations. Multicollinearity was checked using Variance Inflation Factor (VIF), and all VIF values were below 2, indicating an absence of multicollinearity.

## 3. Results

### 3.1. Demographic Data Analyses

The general characteristics of the study participants are presented in Table 1. A total of 400 participants were involved in this study; however, 8 participants were excluded for not completing the questionnaires. The mean age of the participants was 28.69 years (S.D. = 7.61), with an age range of 18 to 59 years. Approximately 63.5% (*n* = 249) of the participants were female, and 83.7% (*n* = 328) were aged 35 years or younger. Additionally, 91.1% (*n* = 357) of the participants were non-degree holders. More than half of the participants (55.6%, or *n* = 218) reported current or past experience in caring for older adults. Most participants (68.9%, or *n* = 270) indicated that they had known someone with dementia or memory difficulties. However, 85.7% (*n* = 336) reported that they had never had current or past experience caring for older adults with dementia. Furthermore, 64.0% (*n* = 251) of the participants indicated that they had no formal or informal knowledge of dementia. Table 1 summarizes the demographic characteristics of the respondents.

### 3.2. Descriptive Analyses on Knowledge of Ageing and Attitudes Towards Ageing

#### 3.2.1. The Level of Knowledge of Ageing Among the Study Participants

Knowledge was assessed using Palmore’s Facts on Ageing Quiz (FAQ1), which has a possible score range of 0 to 25. In the current study, participants’ scores ranged from 6 to 21. The results indicated that the public in Kuala Lumpur possesses a moderate level of knowledge about ageing, with a mean score of 13.11 (S.D. = 2.76), as shown in Table 2. Overall, only 5.1% (20 participants) demonstrated high knowledge of ageing, while 88.5% (*n* = 347) displayed a moderate level, and 6.4% (*n* = 25) had a low level of knowledge about ageing (see Table 2).

#### 3.2.2. The Level of Attitudes Towards Ageing Among the Study Participants

Attitudes towards ageing were measured using Kogan’s Attitudes of Older People (KAOP). The findings indicate that the public in Kuala Lumpur holds a slightly positive attitude towards ageing, with a mean score of 126.01 (S.D. = 9.46). Participants’ attitudes ranged from 93 to 150, reflecting a spectrum from slightly negative to positive attitudes. Table 3 presents the frequency distribution based on these scores. The results revealed that 22.4% (*n* = 88) of participants exhibited slightly negative attitudes towards ageing, while 77.5% (*n* = 304) displayed slightly positive to positive attitudes (see Table 3).

The total score for the positive attitudes (KAOP + ve) and negative attitudes (KAOP − ve) towards ageing subscales is 102. A higher score on the positive attitude subscale indicates more favorable views on ageing, while a higher score on the negative attitude subscale reflects more negative perceptions. The results revealed that participants had a mean score of 65.28 (S.D. = 6.88) for positive attitudes, with scores ranging from 33 to 86. In contrast, the mean score for negative attitudes was 58.20 (S.D. = 7.68), with a score range of 31 to 78.

#### 3.2.3. Moderation Regression Analyses on the Relationship Between Knowledge of Ageing (FAQ1) and Positive Attitudes Towards Ageing (KAOP + ve)

A hierarchical multiple regression was employed to evaluate how the moderating variable influences the relationship between knowledge of ageing and attitudes towards ageing. Specifically, we explored the interaction effect of the moderator and knowledge of ageing to determine whether this effect significantly predicts attitudes towards ageing.

##### Knowledge of Ageing and Exposure to Older Adults

To determine whether exposure to older adults moderates the relationship between knowledge of ageing and attitudes towards ageing, a hierarchical multiple regression was conducted. In the first step, two variables were included: (1) knowledge of ageing (FAQ) and (2) experience caring for older adults. These variables explained a significant portion of the variance in positive attitudes towards ageing, R^2^ = 0.48, F(2, 384) = 9.77, *p* < 0.001. In the second step, the interaction term between knowledge of ageing and exposure to older adults was added, but it did not significantly increase the variance explained by the model. Consequently, there was no significant interaction between knowledge of ageing and exposure to older adults, and no moderation analysis was performed.

##### Knowledge of Ageing and Exposure to Someone with Dementia

To examine whether exposure to someone with dementia moderates the relationship between knowledge of ageing and attitudes towards ageing, a hierarchical multiple regression was conducted. In the first step, two variables were included: (1) knowledge of ageing (FAQ) and (2) knowing someone with dementia. These variables explained a significant portion of the variance in positive attitudes towards ageing, R^2^ = 0.45, F(2, 384) = 9.12, *p* < 0.001. In the second step, the interaction term between knowledge of ageing and exposure to someone with dementia also significantly predicted positive attitudes towards ageing (*p* < 0.001), indicating that exposure to someone with dementia functioned as moderator of the relationship between knowledge of ageing and attitudes towards ageing.

A moderated multiple regression analysis was conducted using PROCESS [45] to evaluate the increase in explained variance by adding an interaction term between knowledge of ageing and having experience with someone with dementia to the main-effects model [46]. The results indicated that knowledge of ageing significantly predicted positive attitudes towards ageing, b = 0.56, *p* < 0.001, 95% CI [0.32, 0.79]. Next, experience knowing someone with dementia did not significantly predict positive attitudes towards ageing, *p* < 0.001, 95% CI [0.32, 0.79]. In contrast, having experience with someone with dementia did not significantly predict positive attitudes towards ageing, b = −0.55, *p* = 0.462, 95% CI [−2.02, 0.92]. The interaction between knowledge of ageing and experience with someone with dementia was significant, b = −0.54, *p* = 0.017, 95% CI [−0.98, −0.097], indicating that the impact of knowledge of ageing on positive attitudes towards ageing depended on whether or not individuals had experience with someone with dementia. This exposure moderated the effect of knowledge of ageing on attitudes towards ageing, demonstrated by a statistically significant increase in total variation explained of 1.1%, R^2^ = 0.011, F(1, 383) = 4.34, *p* = 0.038.

**Figure 1:**
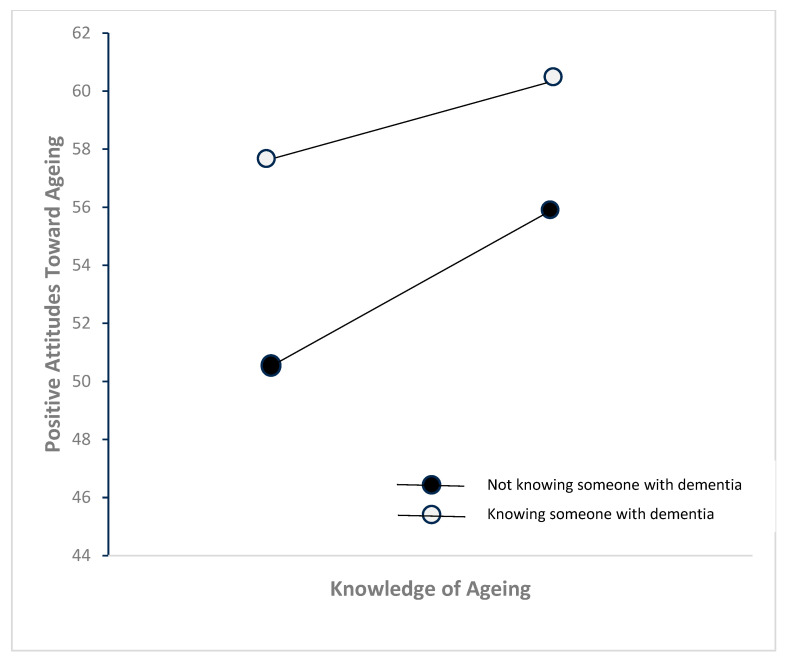
The moderation effect of knowing someone with dementia on the relationship between knowledge of ageing and attitudes towards ageing.

Further analysis of simple slopes revealed a statistically significant positive linear relationship between knowledge of ageing and positive attitudes towards ageing for those without exposure to someone with dementia, b = 0.72, *p* < 0.001, 95% CI [0.41, 1.03], but not among participants who had exposure to someone with dementia, *p* < 0.001, 95% CI [0.41, 1.03]. However, this relationship was not observed among participants with exposure to someone with dementia, b = 0.18, *p* = 0.383, 95% CI [−0.23, 0.59]. Table 4 illustrates the moderation effect of knowing someone with dementia on the relationship between knowledge of ageing and attitudes towards ageing. The findings from the moderation analysis are illustrated in Figure 1.

##### Moderation Effect of Experience Taking Care of Older Adults with Dementia

To evaluate whether exposure to someone with dementia moderates the relationship between knowledge of ageing and attitudes towards ageing, a hierarchical multiple regression was conducted. In the first step, two variables were included: (1) knowledge of ageing (measured by the FAQ) and (2) caring for someone with dementia. These variables accounted for a significant portion of the variance in positive attitudes towards ageing (R^2^ = 0.44, F(2, 384) = 8.86, *p* < 0.001). Additionally, the interaction term between knowledge of ageing and exposure to someone with dementia significantly predicted positive attitudes towards ageing (*p* < 0.001), indicating that exposure to someone with dementia functioned as moderator of the relationship between knowledge of ageing and attitudes towards ageing (*p* < 0.001), suggesting that exposure to someone with dementia acts as a moderator in the relationship between knowledge of ageing and attitudes towards ageing.

A moderated multiple regression was conducted to evaluate how the inclusion of an interaction term between knowledge of ageing and experience caring for someone with dementia affected the variation explained by the main-effects model. The results indicated that knowledge of ageing significantly predicted positive attitudes toward ageing, with b = 0.54, *p* < 0.001, 95% CI [0.31, 0.77]. Next, experience knowing someone with dementia did not significantly predict positive attitudes towards ageing, <0.001, and a 95% confidence interval of [0.31, 0.77]. In contrast, experience knowing someone with dementia did not significantly predict positive attitudes toward ageing, with b = −1.21, *p* = 0.303, and a 95% confidence interval of [−3.51, 1.09]. However, the interaction between knowledge of ageing and experience caring for someone with dementia was significant, with b = −0.87, *p* = 0.002, and a 95% confidence interval of [−1.41, −0.33]. This suggests that the effect of the knowledge of ageing on positive attitudes toward ageing depended on experience with caring for older adults with dementia. Specifically, experience caring for older adults with dementia moderated the effect of knowledge of ageing on positive attitudes, as demonstrated by a statistically significant increase in the total variation explained of 1.6%, with R^2^ = 0.016, F(1, 382) = 6.68, and *p* = 0.001.

**Figure 2:**
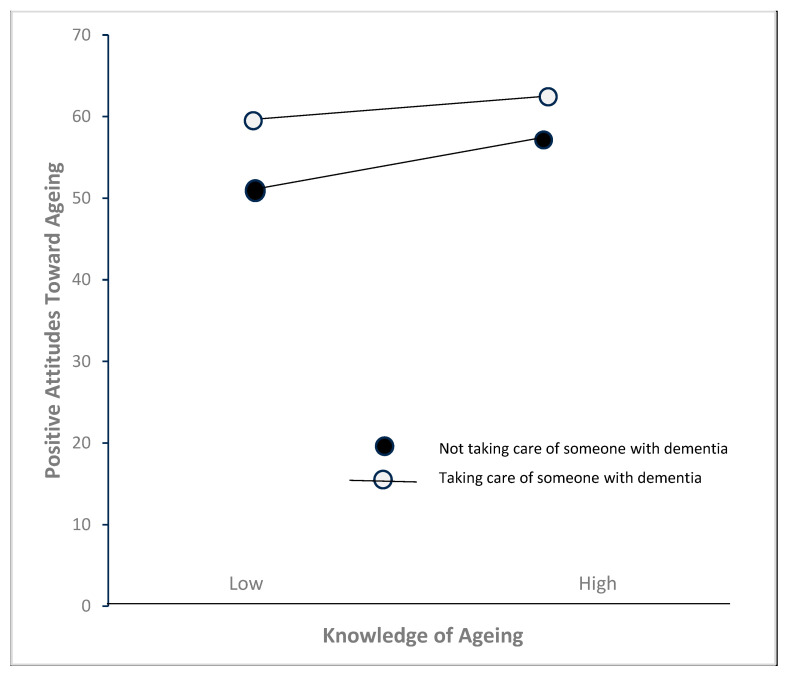
Moderation effect of experience with taking care of older adults with dementia on the relationship between knowledge of ageing and attitudes towards ageing.

Simple slopes analysis indicated a statistically significant positive linear relationship between knowledge of ageing and attitudes towards ageing for participants without exposure to someone with dementia, b = 0.654, *p* < 0.001, 95% CI [0.38, 0.92], but not among participants who had exposure to someone with dementia, <0.001, 95% CI [0.38, 0.92]. In contrast, no significant relationship was found among participants with exposure to someone with dementia, b = −0.22, *p* = 0.48, 95% CI [0.82, −0.38]. Table 5 illustrates the moderating effect of caregiving for older adults with dementia on the relationship between knowledge of ageing and attitudes towards ageing. Figure 2 illustrates the findings related to the moderation analysis.

## 4. Discussion

The current study first aimed to assess the knowledge and attitudes toward ageing among the public in Kuala Lumpur. Second, we examined the influence of sociodemographic variables on knowledge and attitudes toward ageing. Finally, we investigated whether exposure to older adults, particularly those with dementia, moderated the relationship between knowledge of ageing and attitudes toward ageing. The results indicated that the public in Kuala Lumpur possessed an average level of knowledge and slightly positive attitudes toward ageing. Previous studies have often overlooked public perceptions regarding knowledge and attitudes toward ageing, resulting in limited comparisons with other populations. However, the current findings align with earlier research in Malaysia that focused on healthcare students. Most healthcare students in those studies demonstrated a moderate knowledge of ageing [46], with good knowledge among medical and nursing students [47]. This suggests that even though most participants in the current study were not employed in health-related careers, their enrollment in health programs allowed them to achieve a moderate understanding of ageing, similar to populations with formal education in health and ageing topics. Thus, it appears that the majority of participants in Kuala Lumpur have developed a reasonable understanding of ageing. Furthermore, these findings are consistent with those of Cummings [48], who found that the majority of participants from the United States also exhibited a moderate level of knowledge about ageing.

The current findings align with those of Singh et al. [49], who reported slightly positive attitudes among healthcare students in Malaysia. Additionally, Al Ghailani et al. [50] found that doctors and medical students in Oman exhibited moderate attitudes towards ageing. In contrast, the current findings contradict those of Damulak et al. [51], who indicated negative attitudes towards ageing among healthcare students in Malaysia. Despite these mixed results, the current study suggests that attitudes towards ageing among the public in Kuala Lumpur are slightly positive, even though only a small percentage of participants were engaged in health-related fields.

This finding may be influenced by the demographic background of the participants, with the majority categorized as young adults aged 18 to 35 years (*n* = 328) and only a small number classified as middle-aged adults between 36 and 59 years old (*n* = 64). Although no significant differences in knowledge about ageing were found between the age groups, the majority of young adults exhibited a moderate level of knowledge compared to their middle-aged counterparts. This difference may have contributed to the overall mean score in the current study. One possible explanation is that young adults benefit from advancements in technology. A study by Latha et al. [52] suggests that digital media effectively disseminate awareness of mental health. Furthermore, younger adults have grown up with technology, while older adults were introduced to it later in life [53]. This may fundamentally change how individuals think, seek knowledge, and interact with others [53]. Therefore, the advancement of technology may enhance awareness of ageing among young adults, who make up the majority of participants in this study. Consequently, the imbalance in age distribution within this study needs to be addressed and explored further in future research.

The current study also aimed to investigate how exposure to older adults moderates the knowledge of ageing and attitudes towards ageing. Exposure was categorized into three types: (1) caring for older adults, (2) caring for older adults with dementia, and (3) knowing someone with dementia. It was expected that this exposure would influence the relationship between knowledge of ageing and attitudes towards ageing. The results indicated that there was no significant moderating effect of general experience with caring for older adults. However, the hypothesis received partial support, as it was found that both (1) caring for older adults with dementia and (2) knowing someone with dementia did moderate the relationship between knowledge of ageing and positive attitudes towards ageing. This suggests that the combination of knowledge of ageing and exposure to older adults with dementia affects the level of positive attitudes towards ageing.

The findings also indicate that individuals who have been exposed to older adults with dementia tend to develop more positive attitudes toward ageing, especially when they possess knowledge about ageing. Generally, those with both exposure to older adults with dementia and a high level of knowledge about ageing are more likely to exhibit positive attitudes. Regardless of their level of knowledge, individuals with exposure to older adults with dementia showed more favorable attitudes than those without such exposure. Furthermore, those who had exposure and a high level of knowledge about ageing reported even more positive attitudes. Although earlier findings in this study suggested that exposure to older adults with dementia was linked to less favorable attitudes toward ageing, this relationship is moderated by the interaction between knowledge of ageing and exposure to older adults.

This finding indicates that the relationship between knowledge and attitudes toward ageing may be influenced by the level of exposure to older adults with dementia. Specifically, exposure to these individuals can lead to more positive attitudes toward ageing, provided that caregivers possess an appropriate level of knowledge about the ageing process. By enhancing awareness and understanding of ageing, caregivers’ positive attitudes toward those with dementia can be sustained. Accurate information about ageing can help challenge and change negative stereotypes, particularly among those who have had negative experiences with older adults with dementia. This aligns with the idea that fostering quality relationships with older adults can promote more favorable attitudes [54].

Interestingly, the current study found that contact with individuals with dementia weakened the positive relationship between knowledge of ageing and positive attitudes towards ageing. This finding aligns with previous research suggesting that caregiving for individuals with dementia can involve a significant emotional burden, stress, and even negative perceptions due to the cognitive and behavioral challenges presented by dementia [32,33]. Such experiences may create emotional exhaustion, which in turn could mitigate the otherwise beneficial effects of knowledge. Therefore, it is not simply the presence of contact that matters but also the quality and emotional context of that interaction.

Additionally, the findings of the current study provide empirical support for the contact hypothesis [34], while also highlighting its complexity in the context of ageing and dementia. Consistent with previous research by Teater and Chonody [35] and Steward [36], the results show that intergroup contact—in this case, between younger individuals and older adults—can influence attitudes toward ageing. However, echoing the cautionary findings of Drury et al. [25], this study underscores that not all contact yields uniformly positive outcomes. Specifically, the moderation analysis revealed that experience caring for older adults with dementia significantly moderated the relationship between knowledge of ageing and positive attitudes toward ageing. While greater knowledge of ageing was associated with more positive attitudes in participants without caregiving experience, this relationship was not observed in those who had cared for someone with dementia. This suggests that the quality and nature of the contact matter greatly; exposure to older adults with dementia, often in contexts involving stress, dependency, or cognitive decline, may introduce emotional or practical burdens that counteract the benefits of knowledge-based understanding. In line with Allport’s theory [34], this highlights the importance of supportive conditions, such as equal status, cooperation, and positive interdependence, for intergroup contact to reduce prejudice. Therefore, although exposure to older adults with dementia has the potential to shape attitudes, it may not do so positively unless the context of the interaction allows for mutual respect and meaningful engagement.

The current study suggests that improving knowledge about ageing can lead to better outcomes. This aligns with Allport’s [34] contact hypothesis, which posits that increased exposure to an out-group fosters more positive attitudes and reduces prejudice. In this context, the in-group consists of younger individuals, while the out-group is older adults. Continuous interaction with the out-group, particularly through caregiving experiences with older adults, may influence attitudes toward ageing. However, this study’s initial findings contradict Allport’s theory [34]. They revealed that individuals who care for older adults with dementia exhibit less favorable attitudes toward ageing compared to those without such experience. This disparity in attitudes may be better understood through the quality of the caregiver–patient relationship. Caregivers often face significant challenges when caring for older adults with dementia, requiring them to prepare mentally and emotionally for various personality and behavioral issues. These challenges can make caregivers susceptible to high levels of burden, psychological distress, social isolation, physical health problems, and financial strain [33,34]. Thus, this study suggests that regular contact with older adults is insufficient to explain attitude formation without considering the quality of that contact, particularly in cases involving dementia or cognitive decline, such as memory loss. The current findings suggest a new research direction that should investigate the impact of knowledge about ageing, the quality of ageing experiences, and attitudes toward ageing.

## 5. Conclusions, Recommendations, and Practical Implications

This study explored the relationship between knowledge of ageing, exposure to older adults with dementia, and attitudes toward ageing among adults in Kuala Lumpur. The current study involved 392 participants aged between 18 years old and 59 years old. The results indicate that knowledge of ageing is associated with more positive attitudes and that exposure to older adults with dementia moderates this relationship. Specifically, knowledge about ageing appears to play a protective role, particularly in contexts involving challenging contact, such as caregiving for individuals with dementia.

Moreover, the findings revealed that male participants had more knowledge of ageing than female participants. Younger participants showed more positive attitudes towards ageing than middle-aged participants. Those without experience taking care of older adults with dementia showed more positive attitudes than those who had experience taking care of older adults with dementia. Additionally, experience taking care of older adults is uniquely predicted the attitudes towards ageing. Based on further analysis, the exposure towards older adults that comprised the experience taking care of older adults with dementia and know someone with dementia moderated the effect of knowledge on ageing and attitudes towards ageing. In the presence of knowledge, those with exposure towards older adults with dementia showed more positive attitudes towards ageing than those without experience.

Additionally, our study found that individuals with higher levels of knowledge and exposure to older adults developed more positive attitudes compared to those with less knowledge. This suggests that the interaction between knowledge and exposure is crucial in fostering positive attitudes towards ageing. To leverage this finding, we recommend organizing educational visits for students to older adult care centres to increase their interaction with older adults. Hosting more social events, such as volunteer opportunities and community engagement activities, particularly with the ageing population and those with dementia, can further promote positive attitudes

However, the overall variance explained by the moderation models was small, suggesting that other factors beyond knowledge and exposure significantly influence attitudes toward ageing. These findings highlight the importance of educational initiatives aimed at increasing knowledge of ageing and improving the quality of contact experiences with older adults, especially those living with cognitive impairments. Future research using longitudinal designs is needed to clarify causal relationships and to further explore how the quality of intergenerational interactions may strengthen or weaken the association between knowledge and attitudes towards ageing.

The findings of this study highlight important practical implications regarding the moderating effect of exposure to older adults with dementia on attitudes towards ageing. Specifically, the results suggest that experience caring for someone with dementia moderates the relationship between knowledge of ageing and positive attitudes towards ageing. While increased knowledge of ageing was generally associated with more positive attitudes towards ageing, this relationship was weaker among those with exposure to individuals with dementia. The interaction effect showed that for individuals without exposure to someone with dementia, higher knowledge of ageing significantly predicted more positive attitudes, but this effect was not present for those with caregiving experience. These findings imply that exposure to dementia may influence how people perceive ageing, potentially altering the positive impact that knowledge of ageing typically has on attitudes. In practical terms, this suggests that interventions aimed at improving attitudes towards ageing may need to consider the caregiving experience, as individuals with exposure to dementia may require tailored approaches to foster more positive perceptions of ageing. Additionally, understanding the moderating effect of caregiving for those with dementia can inform public health strategies and caregiver support programs, encouraging a more nuanced approach to ageing education.

Additionally, the findings suggest important directions for public policy and educational programming. Efforts to foster positive attitudes towards ageing should not solely focus on increasing knowledge but also emphasize enhancing the quality of intergenerational interactions. Programs that promote structured, positive contact experiences such as service-learning projects, dementia-friendly community initiatives, and targeted educational interventions, could be particularly effective. Policymakers should also consider integrating dementia education into public health campaigns to prepare individuals for realistic, empathetic, and supportive engagement with older adults, particularly those living with dementia.

## 6. Limitations of This Study

This study is notable as the first in Malaysia to examine the moderating effect of knowledge and attitudes towards ageing among the public in Kuala Lumpur. It found that exposure to older adults influences the relationship between knowledge of ageing and individual attitudes towards ageing. Consequently, this research lays the groundwork for future investigations into the public’s knowledge and attitudes regarding ageing in Malaysia. However, several limitations affect the findings of this study.

The first limitation is the lack of ethnic information in the demographic section, which may restrict representation of the diverse adult population in Kuala Lumpur. Malaysia’s multicultural landscape means that different ethnic groups may hold varying perspectives on the ageing population, potentially impacting their knowledge and attitudes towards ageing. Previous research has highlighted the significant role that ethnicity and cultural differences play in shaping attitudes towards ageing [43].

Additionally, this study focused exclusively on the urban population of Kuala Lumpur, limiting the generalizability of the findings to the broader Malaysian population. Different localities may exhibit varying levels of awareness and attitudes toward ageing, and urban residents may have greater access to information and resources than those in rural areas. Therefore, the results should be interpreted with caution.

Another limitation is that contact with older adults was assessed using a simple yes or no response, without evaluating the quality of that contact. The nature of interactions, whether positive or negative, could significantly influence individuals’ attitudes toward ageing. The lack of assessment regarding the quality of contact may have constrained the findings of this study.

Furthermore, it is important to note that while the moderation effects observed in this study were statistically significant, the additional variance explained by the interaction terms was relatively low (1.1% and 1.6%). This suggests that although exposure to older adults with dementia plays a role in moderating the relationship between knowledge and attitudes toward ageing, other unexamined factors likely contribute more substantially to attitudes towards ageing. Future research should consider incorporating additional variables, such as empathy, personal beliefs about ageing, and quality of intergenerational interactions, to better explain variance in attitudes.

Additionally, the FAQ questionnaire-reported alpha coefficient (α = 0.28) is low, suggesting a lack of internal consistency within the scale. While external validity may still be defensible in the context of the findings, this limitation should be acknowledged. Future research should aim to improve the internal reliability of the instrument, possibly through revisions to the questionnaire items or further reliability testing or alternative scales with stronger psychometric properties.

Self-selection bias may have influenced the study results. Participants with a pre-existing interest or positive disposition toward ageing issues may have been more inclined to volunteer, potentially limiting generalizability to the broader public. The cross-sectional design restricts the ability to infer causal relationships. It is unclear whether knowledge leads to positive attitudes or whether individuals with more positive attitudes seek more knowledge about ageing.

Additionally, this study recruited participants solely from Kuala Lumpur, an urban and highly developed setting. As a result, the findings may not be generalizable to rural populations, who may have different levels of exposure to older adults, different cultural perceptions of ageing, and more limited access to educational resources. Future studies should aim to include participants from both urban and rural areas to provide a more comprehensive understanding of knowledge and attitudes towards ageing across diverse settings.

Although multicollinearity was assessed and VIF values were within acceptable ranges (<2), the low increase in R^2^ in the moderation models (1.1% and 1.6%) suggests that other unmeasured factors may play a more substantial role in shaping attitudes towards ageing. Longitudinal or experimental designs could help to more robustly test these relationships. An additional limitation of this study is the presence of missing responses for some questionnaire items. Although mean substitution was used to handle minor missing data, this approach may introduce bias and should be considered when interpreting the findings.

## Figures and Tables

**Table 1:** Demographic characteristics of respondents (N = 392).

Variables	*n*	(%)	Mean (S.D.)
Age			28.69 (7.61)
Age group			
Young adults (18–35)	328	83.7	
Adults (36–59)	64	16.3	
Gender			
Male	141	36.0	
Female	249	63.5	
Education			
Degree graduate	31	7.9	
Non-degree graduate	357	91.1	
Do you have current or past experience taking care of the elderly (e.g., parents, relative or close contact)?			
Yes	218	55.6	
No (no/unsure)	170	43.4	
Do you know someone with dementia or has poor memory ability?			
Yes	117	29.8	
No	270	68.9	
Do you have current or past experience of taking care of older adults with dementia (e.g., parents, relatives, other contacts)?			
Yes	50	12.8	
No	336	85.7	
Do you have formal or informal knowledge of dementia disease?			
Yes	137	34.9	
No	251	64.0	

**Table 2:** Distribution of participants in relation to the level of knowledge about ageing.

Classification of Knowledge on Ageing	*n*	%
Low (0–8)	20	5.1
Moderate (9–17)	347	88.5
High (18–25)	25	6.4

**Table 3:** Distribution of participants in relation to attitudes towards ageing.

Classifications	Range of Scores	Frequency	Percent
Very negative	34–62.3	0	0
Negative	62.4–90.7	0	0
Slightly negative	90.8–119.1	88	22.4
Slightly positive	119.2–147.5	302	77.0
Positive	147.6–175.9	2	0.5
Very positive	176–204	0	0

**Table 4:** Moderation effect of knowing someone with dementia on the relationship between knowledge of ageing (FAQ1) and positive attitudes towards ageing (KAOP + ve) N = 384.

	*b* Coefficient	*SE*	*t*	*p*	95% CI	
Constant	55.97	2.11	26.47	0.000	51.81	60.13
Knowledge of ageing (FAQ1)	0.72	0.16	4.60	0.000	0.41	1.03
Knowing someone with dementia	6.53	3.45	1.89	0.059	−0.25	13.32
Knowledge of ageing (FAQ1) × Knowing someone with dementia	−0.54	0.26	−2.08	0.038	−1.05	−0.03

Model summary: *R* = 0.24; *R*-sq changed = 0.011, F = 4.34 df 1 = 1 df 2 = 383, *p* = 0.0380.

**Table 5:** Moderation effect of experience taking care of older adults with dementia on the relationship between knowledge of ageing (FAQ1) and positive attitudes towards ageing (KAOP + ve) N = 384.

	*b* Coefficient	*SE*	*t*	*p*	95% CI	
Constant	56.78	1.84	30.85	0.000	53.16	60.40
Knowledge of ageing (FAQ1)	0.65	0.14	4.79	0.000	0.38	0.92
Experience taking care of older adults with dementia	10.21	4.35	2.34	0.020	1.65	18.77
Knowledge of ageing (FAQ1) × Experience taking care of older adults with dementia	−0.87	0.34	−2.58	0.010	−1.53	−0.21

Model summary: *R* = 0.25; *R*-sq changed = 0.02, F = 6.6779 df 1 = 1 df 2 = 382, *p* = 0.01.

## Data Availability

Aggregated data supporting this study’s findings are available upon reasonable request from the corresponding author.
